# Inhibition of circulating dipeptidyl-peptidase 3 by procizumab in experimental septic shock reduces catecholamine exposure and myocardial injury

**DOI:** 10.1186/s40635-024-00638-3

**Published:** 2024-06-07

**Authors:** Bruno Garcia, Benoit ter Schiphorst, Karine Santos, Fuhong Su, Laurence Dewachter, Francisco Vasques-Nóvoa, Estela Rocha-Oliveira, Roberto Roncon-Albuquerque, Theo Uba, Oliver Hartmann, Adrien Picod, Feriel Azibani, Jacques Callebert, Serge Goldman, Filippo Annoni, Raphaël Favory, Jean-Louis Vincent, Jacques Creteur, Fabio Silvio Taccone, Alexandre Mebazaa, Antoine Herpain

**Affiliations:** 1 Experimental Laboratory of the Department of Intensive Care, Université Libre de Bruxelles (ULB), Brussels, Belgium; 2 Department of Intensive Care, Centre Hospitalier Universitaire de Lille, Lille, France; 3 4TEEN4 Pharmaceuticals GmbH, Hennigsdorf, Germany; 4 Laboratory of Physiology and Pharmacology, Université Libre de Bruxelles (ULB), Brussels, Belgium; 5 Cardiovascular R&D Center, Faculty of Medicine, University of Porto, Porto, Portugal; 6 Université Paris Cité, UMR-S 942, INSERM, MASCOT, Paris, France; 7 Department of Biochemistry, Assistance Publique Hôpitaux de Paris, Hôpital Lariboisière, Paris, France; 8 Department of Nuclear Medicine, Hôpital Universitaire de Bruxelles (HUB), Université Libre de Bruxelles (ULB), Brussels, Belgium; 9 Department of Intensive Care, Hôpital Universitaire de Bruxelles (HUB), Université Libre de Bruxelles (ULB), Brussels, Belgium; 10 Department of Anesthesia, Burn and Critical Care, University Hospitals Saint-Louis-Lariboisière, AP-HP, Paris, France; 11 Department of Intensive Care, Saint-Pierre University Hospital, Université Libre de Bruxelles (ULB), Brussels, Belgium

**Keywords:** Sepsis, Angiotensin II, Dipeptidyl peptidase 3, Vasopressors, Renin–angiotensin system, Shock

## Abstract

**Background:**

Dipeptidyl peptidase 3 (DPP3) is a ubiquitous cytosolic enzyme released into the bloodstream after tissue injury, that can degrade angiotensin II. High concentrations of circulating DPP3 (cDPP3) have been associated with worse outcomes during sepsis. The aim of this study was to assess the effect of Procizumab (PCZ), a monoclonal antibody that neutralizes cDPP3, in an experimental model of septic shock.

**Methods:**

In this randomized, open-label, controlled study, 16 anesthetized and mechanically ventilated pigs with peritonitis were randomized to receive PCZ or standard treatment when the mean arterial pressure (MAP) dropped below 50 mmHg. Resuscitation with fluids, antimicrobial therapy, peritoneal lavage, and norepinephrine was initiated one hour later to maintain MAP between 65–75 mmHg for 12 h. Hemodynamic variables, tissue oxygenation indices, and measures of organ failure and myocardial injury were collected. Organ blood flow was assessed using isotopic assessment (^99m^technetium albumin). cDPP3 activity, equilibrium analysis of the renin–angiotensin system and circulating catecholamines were measured. Tissue mRNA expression of interleukin-6 and downregulation of adrenergic and angiotensin receptors were assessed on vascular and myocardial samples.

**Results:**

PCZ-treated animals had reduced cDPP3 levels and required less norepinephrine and fluid than septic control animals for similar organ perfusion and regional blood flow. PCZ-treated animals had less myocardial injury, and higher PaO_2_/FiO_2_ ratios. PCZ was associated with lower circulating catecholamine levels; higher circulating angiotensin II and higher angiotensin II receptor type 1 myocardial protein expression, and with lower myocardial and radial artery mRNA interleukin-6 expression.

**Conclusions:**

In an experimental model of septic shock, PCZ administration was associated with reduced fluid and catecholamine requirements, less myocardial injury and cardiovascular inflammation, along with preserved angiotensin II signaling.

**Graphical Abstract:**

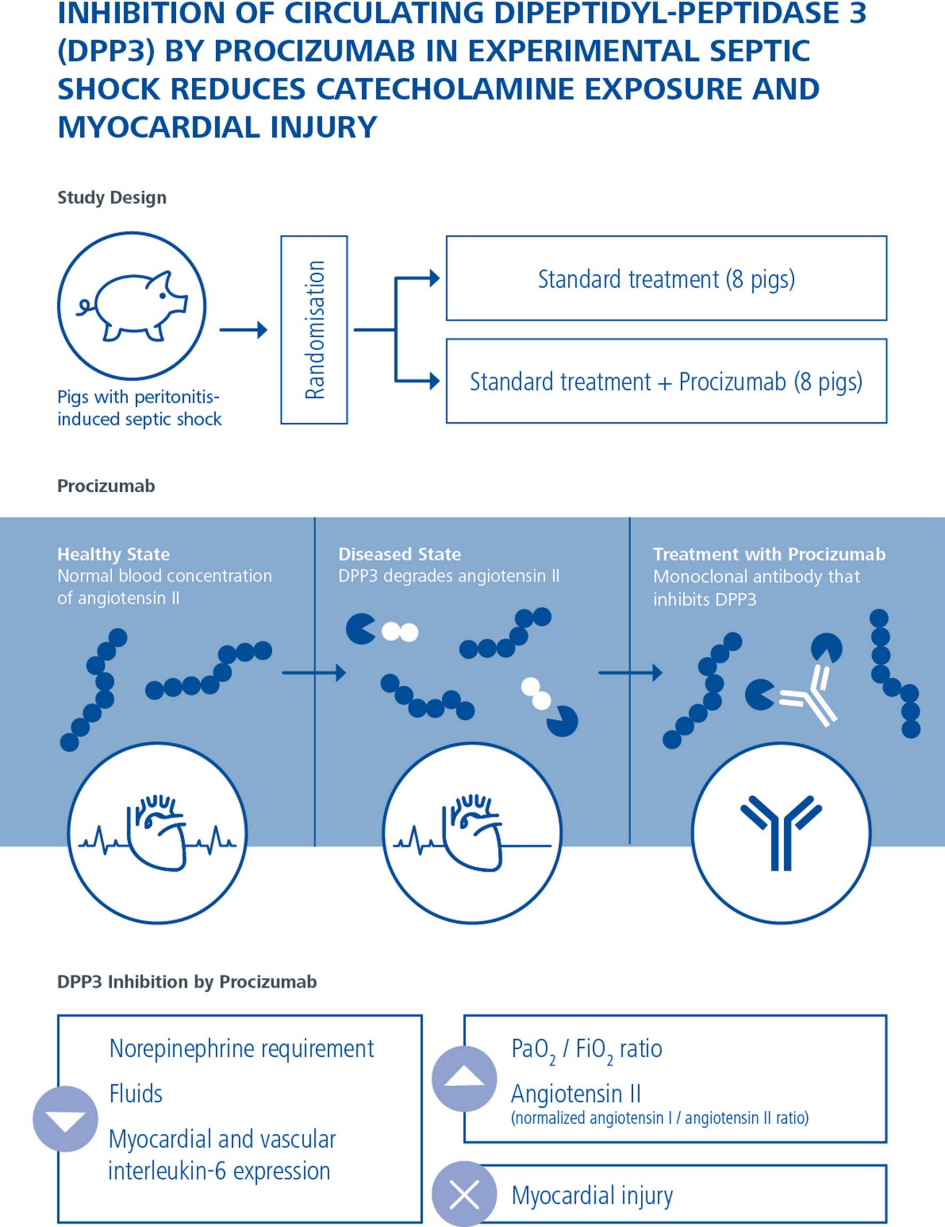

**Supplementary Information:**

The online version contains supplementary material available at 10.1186/s40635-024-00638-3.

## Introduction

Sepsis is a dysregulated host response to infection, resulting in life-threatening organ dysfunction [1]. It represents 20% of all deaths worldwide, making it the primary cause of mortality and responsible for 11 million deaths per year [2, 3].

There have been recent advances in our understanding of the pathophysiology of sepsis, including the role of the renin–angiotensin system (RAS). Under physiological conditions, renin is released in response to a decrease in tissue perfusion or sympathetic activation [4, 5]. Angiotensinogen, produced by the liver, is converted by renin into angiotensin I (Ang I), which is then cleaved into angiotensin II (Ang II). Ang II is the main effector of the RAS and binds to angiotensin II type 1 receptor (AT_1_), inducing an increase in blood pressure through vasoconstriction, enhanced circulating volume, catecholamine release, and improved cardiac contractility [6]. Recently, elevated renin concentrations were observed in patients with catecholamine-resistant vasodilatory shock, correlating with a high Ang I/Ang II ratio, a marker associated with poor prognosis [7, 8]. A defect in Ang II signaling at a tissue level has also been described, with AT_1_ downregulation observed at both vascular and renal levels, which appears to be largely dependent on the activation of inflammatory signaling pathways [9, 10].

The elevation in the Ang I/Ang II ratio could be related to decreased production of Ang II, due to reduced angiotensin-converting enzyme (ACE) activity, or increased degradation of Ang II, either by ACE type 2 or, as recently suggested, by peptidases, such as dipeptidyl peptidase 3 (DPP3) [8, 11–13]. DPP3 is an intracellular aminopeptidase that is ubiquitously expressed and highly conserved in higher animals where it is implicated in antioxidant response. When released into the bloodstream, circulating DPP3 (cDPP3) cleaves various peptides of the RAS, including Ang II [14, 15]. It has been hypothesized that DPP3 is released into the circulation during sepsis, as a result of tissue injury and/or cell death [16, 17], potentially leading to Ang II degradation and further increase in the Ang I/Ang II ratio [12, 18, 19]. In a multinational study involving 585 patients, cDPP3 concentrations at intensive care unit (ICU) admission were independently associated with increased 28-day mortality [18].

These findings suggest that cDPP3 may be of interest both as a biomarker and as a potential target in sepsis. Procizumab (PCZ), a first-in-class humanized monoclonal antibody that specifically binds to and inhibits cDPP3, has been shown to improve cardiac contractility and decrease myocardial oxidative stress in a mouse model of acute cardiac stress [20], and to restore cardiac dysfunction and improve survival in a rodent model of sepsis [21].

We used a clinically relevant large animal model of septic shock induced by peritonitis to investigate the effects of PCZ on hemodynamics, organ perfusion and dysfunction, RAS disturbance, systemic and vascular pro-inflammatory cytokine levels.  Vascular and cardiac angiotensin and adrenergic receptor regulation were also assessed.

## Methods

The open-label, controlled study protocol adhered to the EU Directive (2010/63/EU) for animal experiments and was approved by the local animal ethics committee, Comité Ethique du Bien-Être Animal (protocol number 723N), at the Université Libre de Bruxelles (ULB) in Brussels, Belgium. Experiments were conducted in the Experimental Laboratory of the Department of Intensive Care at ULB (LA1230336), and the ARRIVE guidelines and MQTiPSS recommendations for translational research in sepsis were followed [22, 23].

### Experimental procedure

**Fig. 1:**
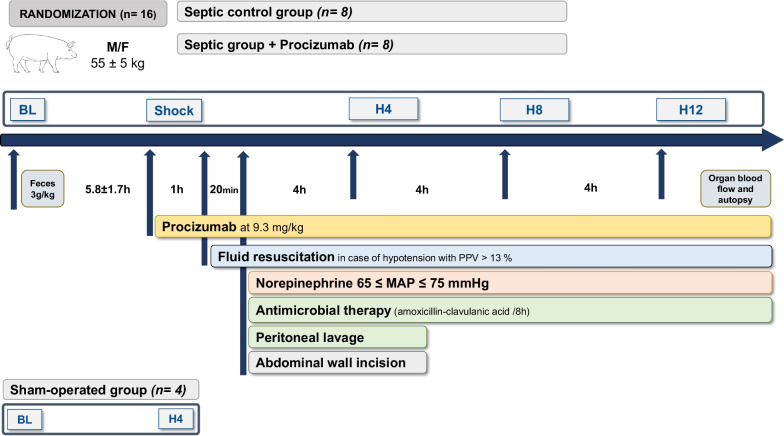
Protocol timeline. Two hours after the end of the instrumentation, baseline measurements were obtained and feces were injected into the peritoneum. Animals were allowed to develop sepsis until a severe hypotensive state arbitrarily set at a mean arterial pressure (MAP) ≤ 50 mmHg (corresponding to the shock time‑point) was reached. According to prior randomization, PCZ was started in the treated group. Severe hypotension (between 45 and 50 mmHg MAP) was left untreated for one hour. Thereafter, fluid resuscitation was started for 20 min. Full resuscitation was then started with norepinephrine, source control, antibiotics and abdominal wall incision. Animals were euthanized 13 h and 20 min after the shock time point

We used an established porcine model of septic shock [24–26] with 16 septic pigs (*Sus scrofa domesticus*, RA-SE Genetics, Belgium), weighing 55 ± 5 kg, and 4 sham-operated. The animals were randomized the day before the experiment to either PCZ administration in addition to standard treatment or standard treatment. The experimental protocol and study time-points are illustrated in Fig. 1 and full details of the experimental procedure are provided in the supplement.

Briefly, after anesthesia and surgery, animals were allowed to stabilize for two hours. The end of this two-hour period was considered the baseline timepoint. Anesthesia was maintained with a continuous inhalation of sevoflurane (at 1.8 to 2.5% alveolar concentration), and analgesia was provided through a continuous infusion of morphine (0.2 to 0.5 mg/kg/h) in combination with rocuronium (1.8 to 2.0 mg/kg/h). Sepsis was then induced by an intraperitoneal instillation of 3 g/kg of autologous feces, previously collected from the animal's cage, via two peritoneal drains. The maintenance infusion rate of balanced crystalloid (Plasmalyte, Baxter, USA) was reduced to 1 mL/kg*h until the animal developed severe hypotension, defined as a mean arterial pressure (MAP) below 50 mmHg (corresponding to the *shock* time-point). Animals were kept in a hypotensive state for one hour to enforce the organ dysfunction. Fluid resuscitation was then started with 10 mL/kg*h of balanced crystalloid (Plasmalyte, Baxter, USA) and 10 mL/kg*h of colloid (Geloplasma, Fresenius Kabi, France) over a period of 20 min, to restore an optimal preload based on an arterial pulse pressure variation (PPV) ≤ 13%. Then, norepinephrine was started, targeting a MAP between 65 and 75 mmHg. At this point, broad spectrum antibiotic with 2 g amoxicillin-clavulanic acid was initiated and repeated 8 h later. In parallel, peritoneal drains were opened to remove peritoneal fluid and peritoneal lavage was performed with the infusion of 1 L of crystalloids, previously warmed to 40 °C, through the peritoneal drains. The abdominal wall was opened surgically (without opening the peritoneum) to limit the increase in intra-abdominal pressure and prevent abdominal compartment syndrome [27]. Fluids were titrated to maintain a PPV ≤ 13% in the case of a decrease in MAP. Full resuscitation with source control, fluids, and norepinephrine was continued for 12 h, with hemodynamic time-points recorded every hour (labeled as H1 to H12) and biological time-points at 4, 8, and 12 h after the start of norepinephrine (labeled as H4, H8, and H12, respectively). Animals were euthanatized under deep anesthesia (no reaction to a pain test, i.e., change in heart rate or blood pressure after nasal septum pinching) with a bolus injection of 40 mL of 0.20 g/mL potassium chloride solution. Tissue samples were collected immediately after euthanasia. Four animals (sham-operated group), which underwent only anesthesia and surgical preparation, were observed for 4 h after baseline until euthanasia and were used as a reference for tissue analysis.

Details concerning biological measurements, PCZ infusion, evaluation of DPP3 activity, analysis of pro-inflammatory cytokines, catecholamine measurements, liquid chromatography–tandem mass spectrometry analysis of the RAS, isotopic renal and ileal perfusion assessment, and protein expression levels of receptors in vascular samples are provided in the supplement (Tables S1, S2).

### Statistical analysis

All analyses were predefined. All data are presented as mean ± standard deviation or median [25–75%] unless otherwise stated. In this exploratory study, given the absence of prior experiments in large animal models, a sample size of 16 animals was deemed sufficient to strike a balance between statistical power and ethical consideration. To take into account the repeated measurements structure of the data, a generalized linear model for repeated measures was applied to examine the differences in all analyzed variables among the groups at the different considered time-points. When the normality was rejected, the analyzed variable was log-transformed to fit the normality requirement. A *p* < 0.05 was considered statistically significant; if significant, a post hoc test was applied for each time-point. For variation in mRNA and protein expression, comparison was performed using Kruskal–Wallis test. Data were analyzed using Prism (GraphPad Software Inc., USA) and R software (R Foundation for Statistical Computing, Vienna, Austria).

## Results

### Septic shock induction

**Fig. 2:**
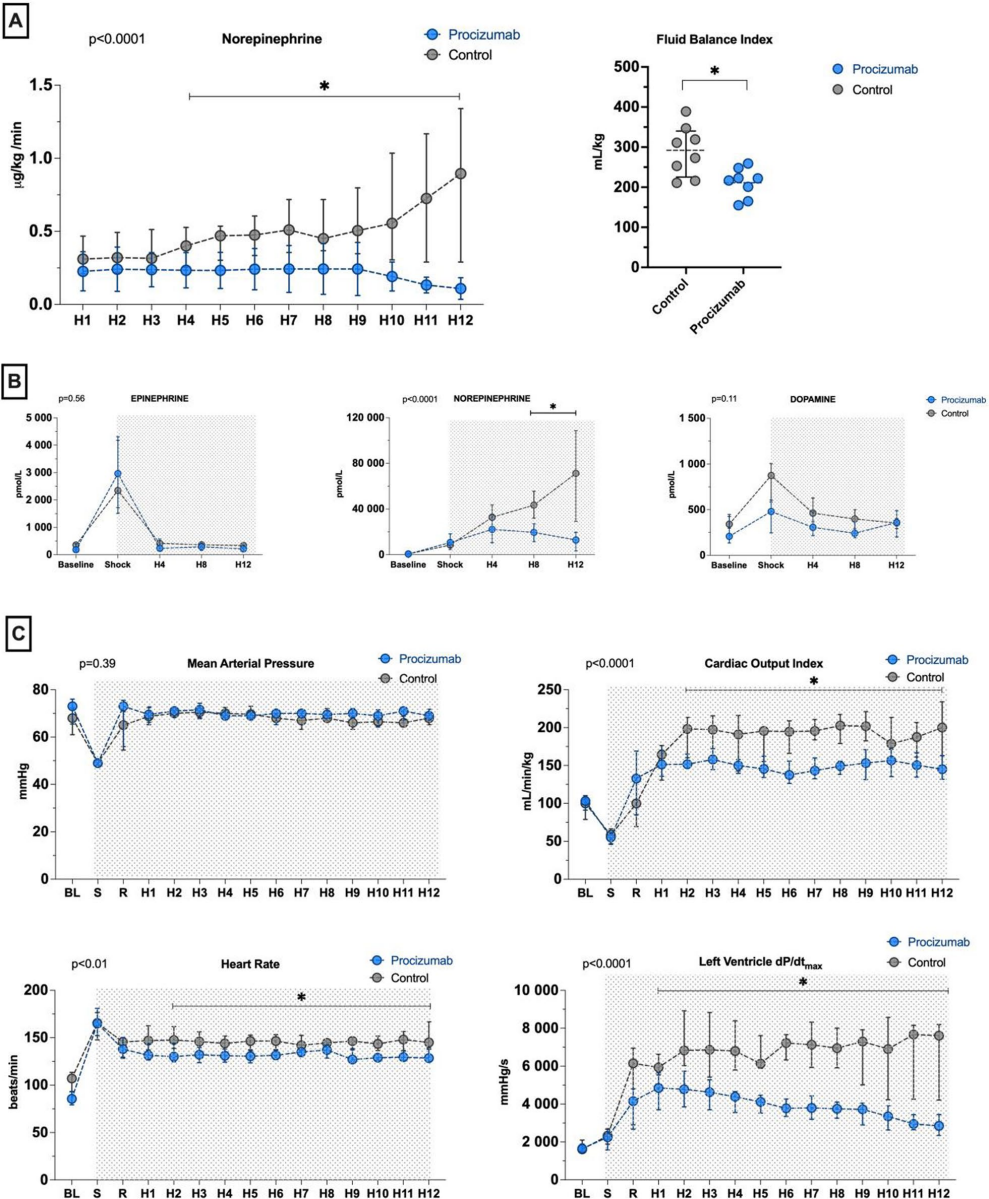
Hemodynamic variables. **A** Norepinephrine base dose during the resuscitation phase. Fluid balance index from shock time point to the end of the experiment. **B** Circulating catecholamines. **C** Mean arterial pressure, cardiac output index, heart rate and left ventricle dP/dT_max_. Values are expressed as median – interquartile range. BL: baseline; S: shock time-point; R: Resuscitation time-point. PCZ group *n* = 8, septic control group *n* = 8. *P* values for interaction were analyzed using a generalized linear model. *P value < 0.05 between PCZ and septic control group for post hoc interactions

All 16 septic animals developed severe hypotension and tachycardia, with decreased mixed venous oxygen saturation (SvO_2_) and increased veno-arterial CO_2_ partial pressure difference (P(v-a)-CO_2_) at the shock time-point compared to baseline. The mean time to reach the shock time-point was similar in the two groups (6.2 ± 1.6 h for PCZ vs. 5.4 ± 1.7 h for septic control group, *p* = 0.35). There were no statistically significant differences in hemodynamic parameters between the treatment groups before PCZ administration (Fig. 2 and Tables 1, S3).

**Table 1:** Biological and oxygenation values in the two groups at the different study timepoints

Variables Median [IQR]		Baseline	Shock	H4	H8	H12	*P* value Interaction
Hematocrit (%)	PCZ Control	27 [24–29] 27 [25–28]	45 [41–48] 43 [41–47]	24 [19–26] 22 [20–30]	24 [20–28] 22 [19–28]	22 [18–23] 20 [19–25]	0.74
Platelets (G/L)	PCZ Control	114 [99–152] 135 [127–147]	100 [53–122] 113 [94–170]	105 [65–121] 96 [64–143]	88 [79–104] 94 [81–99]	85 [69–105] 85 [69–146]	0.88
Albumin (g/L)	PCZ Control	26 [24–31] 27 [20–30]	26 [23–29] 24 [20–28]	9 [7–11] 7 [7–10]	9 [7–10] 7 [6–7]	8 [7–9] 7 [6–7]	0.94
Creatinine (mg/dL)	PCZ Control	1.1 [1.1–1.4] 0.9 [0.8–1]	2.0 [1.7–2.2] 1.7 [1.6–2]	1.6 [1.3–1.7] 1.2 [1.1–1.6]	1.6 [1.3–1.7] 1.2 [1.1–1.6]	1.4 [1.1–1.7] 1.2 [1–1.3]	0.83
Creatinine Clearance (mL/min)	PCZ Control	201 [100–346] 239 [132–516]	61 [44–119] 62 [32–74]	96 [68–137] 80 [53–104]	122 [98–130] 178 [103–258]	121 [109–164] 96 [80–135]	0.32
PaO_2_/FiO_2_	PCZ Control	390 [355–423] 333 [280–383]	352 [310–377] 270 [259–320]	366 [333–380] * 243 [213–293]	310 [298–353] * 193 [178–201]	314 [274–337] * 173 [128–218]	P<0.01
Respiratory system compliance (mL/cmH_2_O)	PCZ Control	37 [34–40] 29 [26–32]	34 [29–37] 27 [23–32]	32 [28–37] 22 [20–26]	29 [23–34] 20 [17–23]	24 [22–31] 20 [15–23]	0.27
Troponin I (ng/L)	PCZ Control	293 [131–1571] 824 [513–1252]				1294 [131–1571]* 3677 [1866–4904]	NA
IL-6 (pg/mL)	PCZ Control	6 [3–9] 6 [4–12]	1129 [795–1157] 1146 [981–1161]	540 [325–1025] 696 [406–1046]	570 [326–861] 677 [424–1067]	466 [309–466] 710 [377–1090]	0.63

Values are expressed as median–interquartile range

*NA* not applicable

*P* value refers to interaction. *post hoc *P* value < 0.05 between PCZ and septic control group

### Global hemodynamics

After fluid resuscitation and during vasopressor therapy, MAP was maintained between 65 and 75 mmHg in all animals (Fig. 2). The median dose of norepinephrine base required to maintain MAP was significantly lower in the PCZ group than the septic control group from the 4th hour of vasopressor therapy until the end of the experiment (interaction *p* < 0.0001, Fig. 2), reaching a median of 0.13 [IQR 0.05–0.17] microg/kg*min in the PCZ group compared to 0.90 [IQR 0.33–1.30] microg/kg*min in the septic control group at H12 (*p* < 0.01). Of note, norepinephrine was weaned in one animal in the PCZ group before the end of the experiment.

Circulating norepinephrine levels were significantly lower in the PCZ group than in the septic control group after resuscitation (interaction *p* < 0.0001); there were no significant differences in circulating epinephrine or dopamine levels (interaction *p* = 0.56 and 0.11, respectively, Fig. 2).

The total cumulative fluid balance (indexed to body weight) between shock time-point and H12 time-point was lower in the PCZ group, with a median value of 220 [IQR 192–229] mL/kg compared to 292 [244–326] mL/kg in the septic control group (*p* = 0.03, Fig. 2).

No differences were observed in pre-load optimization surrogates, with no statistical differences in the left ventricle end-diastolic pressure (LVEDP) (interaction *p* = 0.60), or in the PPV (interaction, *p* = 0.30), between groups throughout the experiment (Figure S2).

cDPP3 plasma activity decreased significantly after PCZ administration compared to the septic control group (Figure S3).

### Cardiovascular system

Heart rate and cardiac index were significantly lower in the PCZ group than in the septic control group from H3 to H12 (interaction *p* < 0.01 and *p* < 0.0001, respectively, Fig. 2). One episode of atrial fibrillation occurred in the septic control group. Left ventricular contractility, assessed by the maximal slope of left ventricle rise in pressure during systolic upstroke (LV dP/dT_max_), increased in both groups with the vasopressor infusion, and was significantly lower in the PCZ group than in the septic control group from H1 to H12 (interaction *p* < 0.0001, Fig. 2).

Myocardial injury was significantly reduced, as shown by lower levels of high sensitivity cardiac troponin I, at H12 in animals receiving PCZ than in septic control animals (*p* < 0.01, Table 1).

**Fig. 3:**
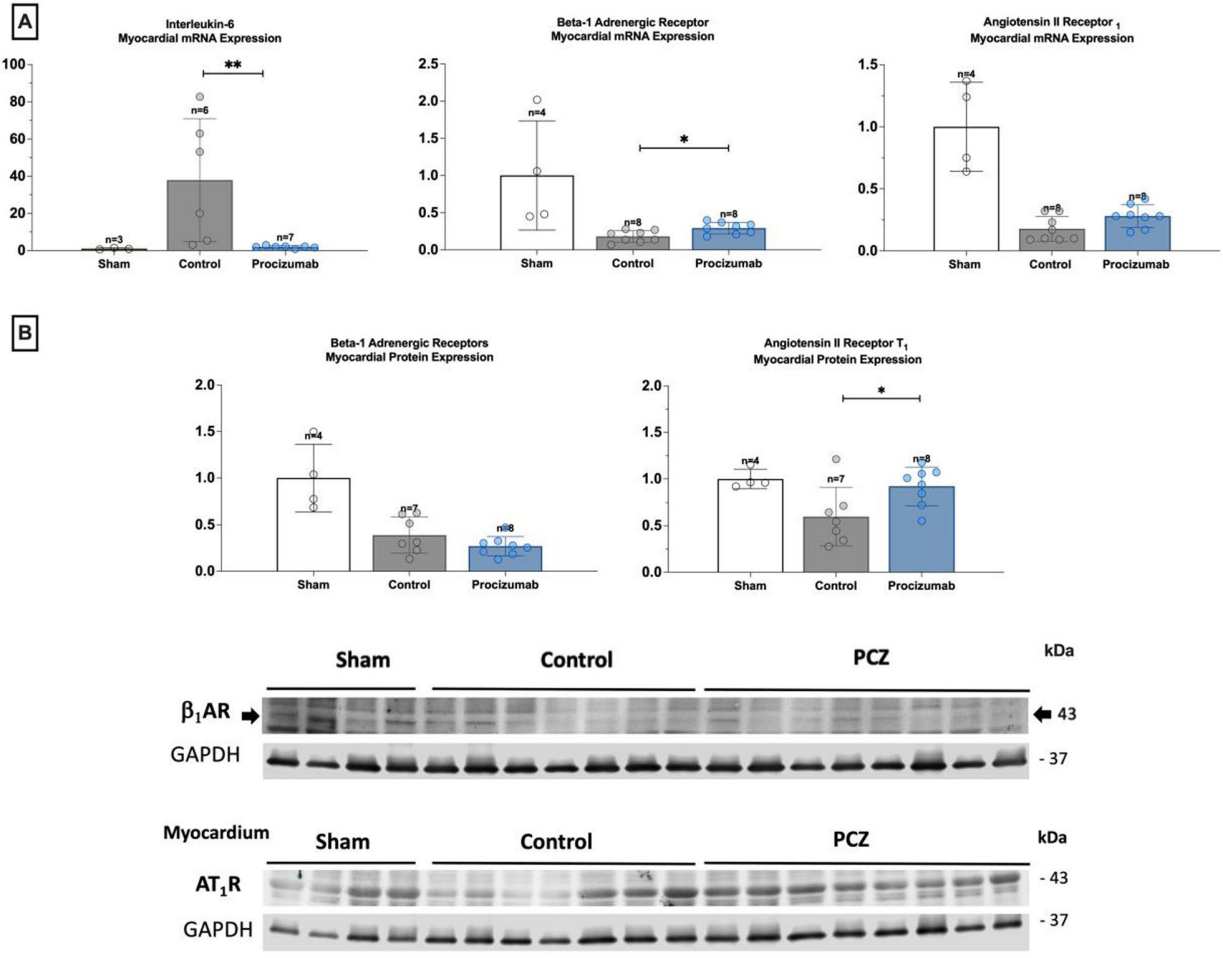
Myocardial IL-6 expression, and myocardial angiotensin and adrenergic receptors. **A** Relative mRNA expression of IL-6 in the myocardium. Relative quantification was achieved using the comparative 2^−ΔΔCt^ method by normalization with the housekeeping gene (ActB‑actin). Results are expressed as relative fold increase above the mean value of relative mRNA expression of the sham group arbitrarily fixed at 1. AT_1_ and b1-AR mRNA expression in the myocardium. Results are expressed as relative fold increase above the mean value of the sham group arbitrarily fixed at 1. **B** AT_1_ and b1-AR protein expression in the myocardium and representative immunoblotting images. Results are expressed as relative fold increase above the mean value of the sham group arbitrarily fixed at 1. Values are expressed as mean and standard deviation. *P value < 0.05 for the Kruskal–Wallis test between PCZ and septic control group

Myocardial inflammatory activity, assessed by IL-6 mRNA expression, was significantly attenuated in the PCZ group (Fig. 3). Beta-1 adrenergic receptor mRNA expression was higher in the PCZ group, while protein expression was similar between groups (Fig. 3).

**Fig. 4:**
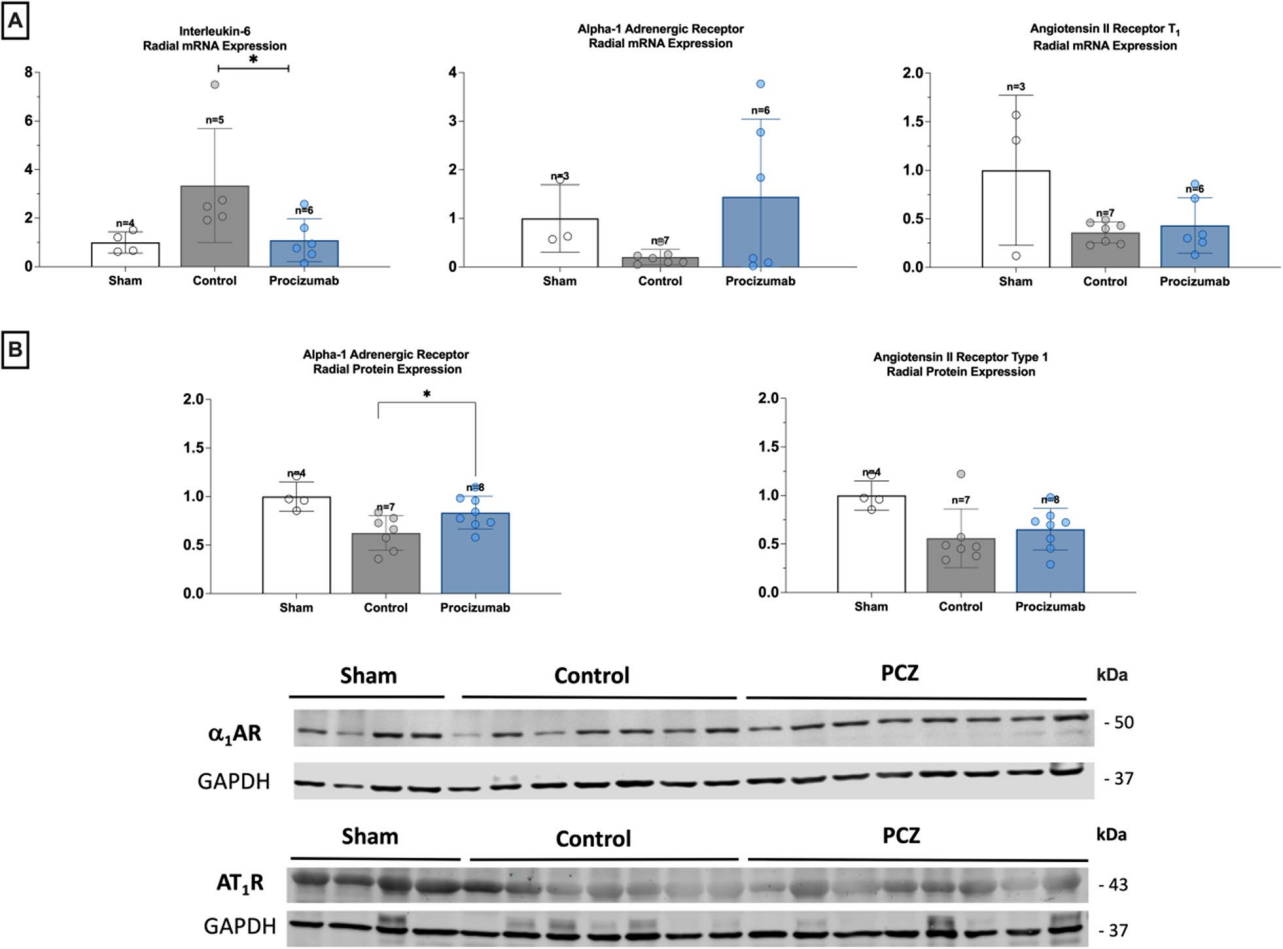
Radial artery IL-6 expression, and radial angiotensin and adrenergic receptors. **A** Relative mRNA expression of IL-6 in the radial arteries. Relative quantification was achieved using the comparative 2^−ΔΔCt^ method by normalization with the housekeeping gene (ActB‑actin). Results are expressed as relative fold increase above the mean value of relative mRNA expression of the sham group arbitrarily fixed at 1. AT_1_ and a1-AR mRNA and protein expression in the radial artery. Results are expressed as relative fold increase above the mean value of the sham group arbitrarily fixed at 1. **B** Alpha-1 adrenergic receptor and AT_1_ protein expression in the radial artery, with representative immunoblotting images. Values are expressed as mean and standard deviation. *P value < 0.05 for the Kruskal–Wallis test between PCZ and septic control group

Vascular inflammatory activity, evaluated by upregulation of IL-6 mRNA levels, was significantly reduced in the radial artery in the PCZ group (Fig. 4). Alpha-1 adrenergic receptor protein expression was higher in the radial artery of PCZ group (Fig. 4), whereas no differences were observed in the other vascular samples.

### Tissue perfusion indices

**Fig. 5:**
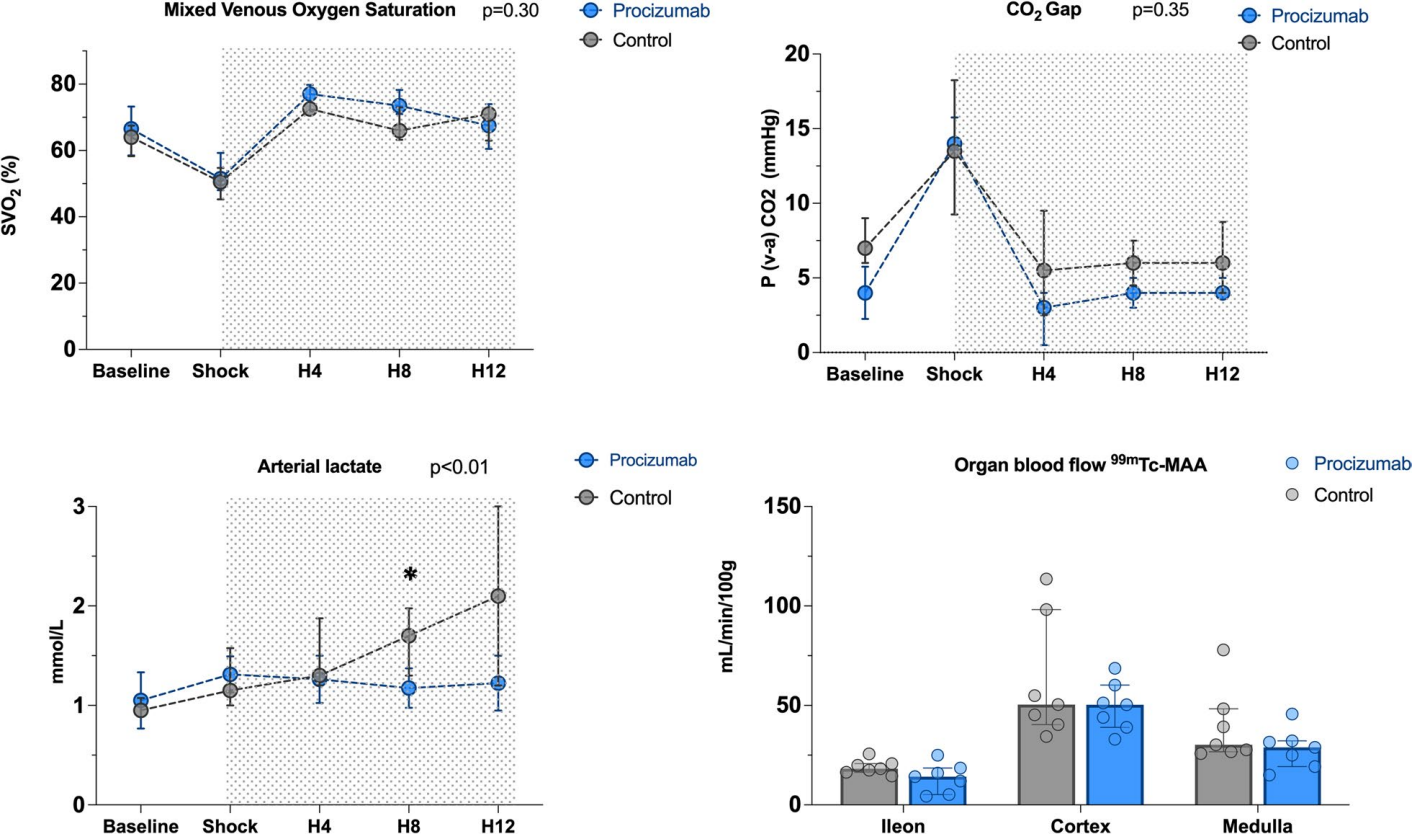
Tissue perfusion indices. Values are expressed as median and interquartile range. *P* values for interaction were analyzed using a generalized linear model. *P value < 0.05 between PCZ and septic control group in case of overall interaction, *n* = 8 for sham, *n* = 8 for PCZ

P-(v-a) CO_2_ and SvO_2_ were restored to and remained at normal values from 4 h after the start of resuscitation; without differences between groups (Fig. 5).

Arterial lactate concentrations were significantly lower in the PCZ than in the septic control group at H8 (interaction *p* < 0.01, Fig. 5). There was no significant difference between groups in regional blood flow, as assessed using 99mTc-macro aggregated albumin in the ileum, the kidney cortex, and the medulla (Fig. 5).

### Pulmonary function, renal system and other parameters

The PaO_2_/FiO_2_ ratio was significantly higher in the PCZ group than in the septic control group from H4 to H12 (interaction *p* = 0.01, Table 1). No difference was observed in respiratory system compliance (Table 1). Creatinine clearance and creatinine levels were similar between groups (*p* = 0.32 and 0.83, respectively, Table 1). Relative changes of creatinine from baseline were similar between groups (interaction *p* = 0.28, Figure S1).

There were no statistically significant differences between groups in aspartate transaminase, alkaline phosphatase, albumin, hematocrit, or platelet count (Tables 1 and S4).

There were no statistically significant differences between the groups in plasma levels of IL-6 (Table 1).

### Renin–angiotensin system

**Fig. 6:**
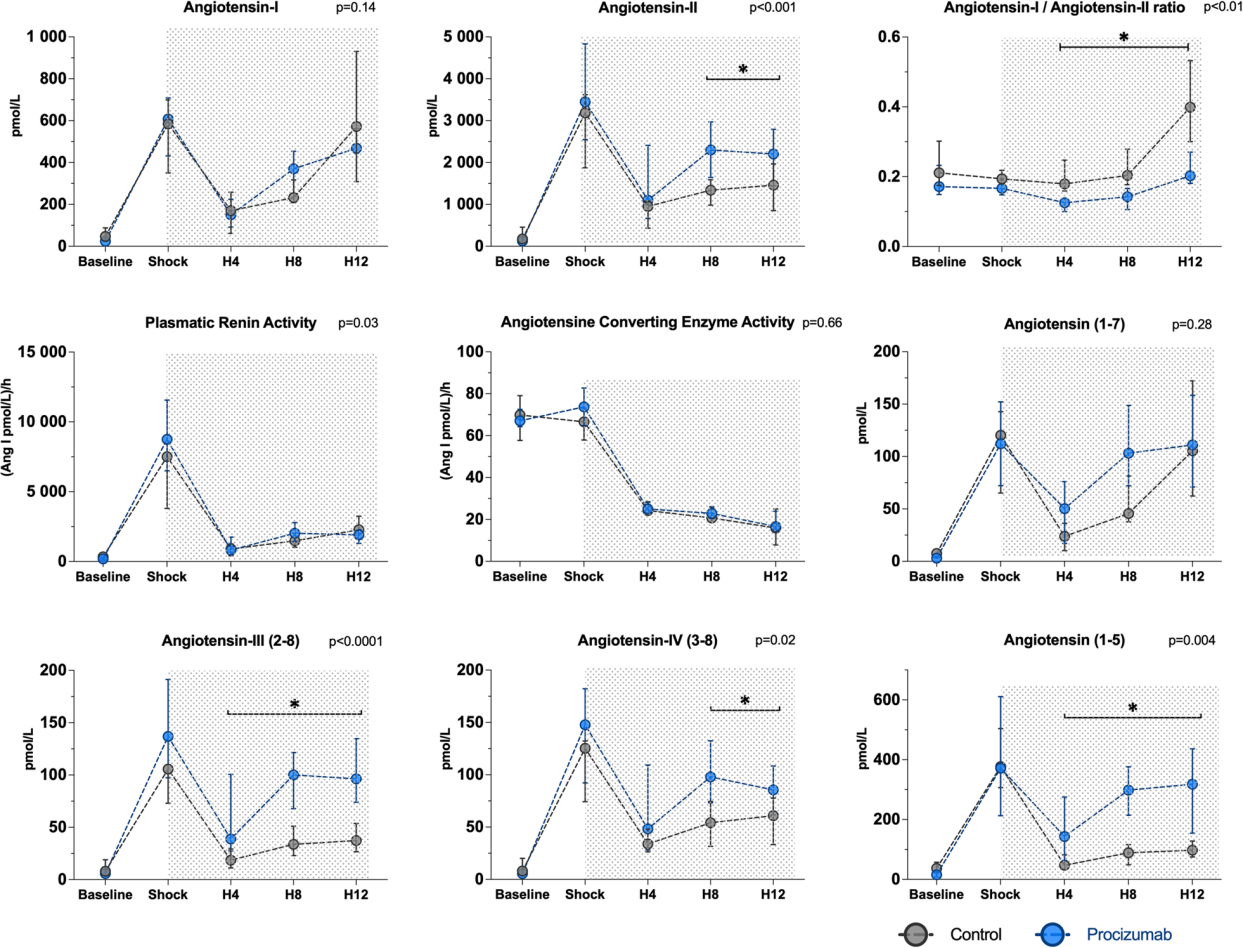
Equilibrium analysis of the renin–angiotensin system. Values are expressed as median and interquartile range. *P* values for interaction were analyzed using a generalized linear model. *P value < 0.05 between PCZ and septic control group

There were no differences between the groups at any time-point in plasma renin activity, ACE activity, or Ang I concentrations. PCZ administration was associated with higher Ang II concentrations compared to the septic control group (interaction *p* < 0.001) since H4 timepoint. The Ang I/Ang II ratio remained stable over time in the PCZ group, but increased significantly in the septic control group from H4 to H12 (interaction *p* = 0.01). PCZ administration was associated with significantly higher Ang III from H4 (interaction *p* < 0.0001), Ang IV from H8 (interaction *p* = 0.02), and Ang-(1–5) from H4 (interaction *p* < 0.01) levels compared to the septic control group (Fig. 6).

Sepsis-induced myocardial AT_1_ receptor protein downregulation was attenuated in the PCZ compared to the septic control group. AT_1_ protein expression was higher in the PCZ group, while mRNA expression was similar between groups. No significant differences were observed in vascular samples (aorta, femoral and radial arteries) regarding angiotensin receptors expression, with exception for lower aortic AT_1_ mRNA expression in the PCZ group (Figures S5–S9).

## Discussion

### Keys findings

In our study, using a fully resuscitated large animal model, we demonstrated that inhibition of DPP3 with PCZ was associated with 1/ Decreased catecholamine and fluid requirements with comparable organ perfusion, as assessed by an isotopic method, 2/ Lower vascular and myocardial inflammation, 3/ Lower myocardial injury and 4/ Better pulmonary function, as reflected by higher PaO_2_/FiO_2_ ratio.

In the PCZ group, the reduction in catecholamine requirement was associated with an increase in Ang II concentration and prevention of the increase in Ang I/Ang II ratio seen in the septic control animals, as assessed by a mass spectrometry method. There was also improved Ang II signaling at the tissue level, with higher AT_1_ protein expression in the left ventricle. Interleukin-6 expression levels were reduced in the vascular and myocardial samples.

### Interpretation of the data and implications of the study findings

The reduction in catecholamine requirements was associated with an increase in the RAS activity, as shown by increased concentration of Ang II and a preserved Ang I/Ang II ratio. Additionally, there was an increase in Ang III, a metabolite of Ang II that can activate AT_1_ and hence increase blood pressure [24]. These findings suggest that PCZ may prevent the reduction in Ang II signaling, thus increasing blood Ang II concentrations. The increase in the Ang I/Ang II ratio was associated with worse outcomes in the ATHOS-3 trial [7].

Although an expected reduction in vasopressor requirement was observed with exogenous Ang II administration in the ATHOS-3 trial [28], some patients did not have this vasoconstrictive response, leading to the hypothesis that peptidases, such as DPP3, could be involved in the lack of response to exogenous Ang II [12, 29]. This provides a rationale for PCZ administration in this setting.

Increased Ang II levels and AT_1_ expression had a beneficial impact on cardiovascular function, with a possible inotropic effect [26] and/or reducing catecholamines exposure on the septic heart [30]. Adrenergic stimulation can increase myocardial oxygen consumption, lead to the downregulation of β-adrenergic receptors, and reverse adrenergic G protein coupling, resulting in impaired myocardial contractility and septic cardiomyopathy, particularly during prolonged administration [31, 32].

In the current study, cardiac output and heart rate were lower in the PCZ group, which may appear contradictory to the increased contractility observed with PCZ in the study by Deniau et al. [21]. In this study, the increased contractility was hypothesized to be related to an increase in blood Ang II concentration in the treated animals. However, norepinephrine was not administered in the animal model used by Deniau et al. [21] whereas, in our study, the strong beta-adrenergic stimulation from the norepinephrine infusion—in both groups—masked any potential moderate inotropic modulation exerted by changes in Ang II signaling. The higher mRNA β1-adrenergic receptor expression in the PCZ group, along with a reduced myocardial IL-6 mRNA expression and myocardial injury compared to the septic control group is consistent with a cardioprotective effect, which aligns with previous observations of PCZ reducing myocardial oxidative stress in mice [20]. Moreover, in a clinical pilot study, the use of Ang II as a primary vasopressor was associated with reduced troponin levels, in line with a possible cardioprotective effect of Ang II signaling during septic shock [33].

Modulation of inflammation is evidenced by a significant reduction in myocardial and radial artery IL-6 mRNA expression in the PCZ-treated animals. Inflammation is one of the mechanisms implicated in the downregulation of angiotensin receptors and the increase in inducible nitric oxide synthase (iNOS) and NO release, both involved in the development of vasoplegia [10, 34, 35]. Increased Ang II concentrations could potentially lead to decreased renin release via a bio-feedback mechanism. Alternatively, Ang II may be converted to anti-inflammatory metabolites, such as Ang 1–7, thereby improving vascular function [36, 37].

Finally, reduction in catecholamine exposure might also contribute to a local reduction in inflammatory response, considering the numerous interactions between immune cells and adrenergic stimulation during sepsis [38], and may improve host defense, as norepinephrine has been shown to dysregulate the immune response and increase bacterial dissemination in experimental models [39].

Other peptides of the RAS were increased in the PCZ group, including Ang III, Ang IV and Ang-(1–5), in line with a study in mice reporting that DPP3 and PCZ administration resulted in regulation of RAS peptides [40]. Increased signaling of the classical and the alternative RAS may contribute to the effects observed in our study [6, 36, 41].

Fluid management was titrated based on the PPV when MAP decreased [42]. The animals in the PCZ group needed less fluid during the experiment. PCZ could potentially prevent fluid overload, which is associated with a worse prognosis in septic shock [43]. The decreased fluid requirement might be due to an increase in vascular tone itself, and/or in AT_1_ signaling [44].

PCZ administration was associated with an improvement in gas exchange, reflected by a higher PaO_2_/FiO_2_ ratio. This was also observed by Wieruszewski et al. in a small retrospective clinical study on exogenous Ang II administration [45] and in a post hoc analysis of the ATHOS 3 trial [46]. This could be related to improved ventilation-perfusion matching, or related to local RAS modulation.

There was no difference in regional blood flow to the kidney and intestine as evaluated using ^99m^Tc albumin micro-aggregates. These findings are consistent with the normalization of tissue perfusion indexes after proper resuscitation in both groups. Arterial lactate was lower in the PCZ group, a result that may be related to higher muscular beta-adrenergic stimulation in the septic control group than to tissue hypoperfusion, given the normal SvO_2_ and P-(v-a) CO_2_ values [47].

Sepsis-associated acute kidney injury, as assessed by creatinine levels, was observed in all animals, with cortical perfusion being higher than medullar perfusion, similar to what has been described in other experimental studies [48]. Impaired Ang II signaling due to decreased AT_1_ expression in the kidney has been documented in sepsis [49]. This was described in a cecal ligature and puncture model, in which sepsis was associated with increased renal blood flow and reduced renal AT_1_ expression [9]. In a large experimental sepsis model performed in sheep, Ang II compared to placebo restored renal blood flow and enhanced creatinine clearance [50]. This effect was not observed in our study. Nonetheless, similar renal function was achieved with a lower fluid balance, which could reduce the risk of venous congestion over an extended observation period [51].

### Limitations

Our study does have several limitations. The open-label study design is an obvious one, even though randomization was performed prior to the start of the experiment, all interventions conducted according to a standardized protocol, with specific targets achieved in the two groups. Additionally, no differences were observed in the PPV or left ventricle end diastolic pressure during the resuscitation phase. The initiation of full resuscitation occurred later in the protocol and was relatively brief, but this timing was chosen to intensify the severity of organ hypoperfusion. However, beneficial effects were observed early in the resuscitation phase, which may be interesting in clinical practice, as the early phase of sepsis is of paramount importance in determining the outcome. Although no quantification of the bacterial load was performed, the progression and severity of sepsis were similar between the two groups. Finally, only mRNA expression was assessed in the tissue samples, given the relatively short duration of the experiments and no time-dependent measurements were made for the sham-operated control group, as this group was used as a reference for the septic tissue samples.

## Conclusions

In this clinically relevant model of septic shock, the addition of PCZ to the standard resuscitation regimen alleviated the hemodynamic alterations, resulting in reduced catecholamines requirements and a lower fluid balance to maintain adequate tissue perfusion. These effects were associated with an increase in circulating Ang II concentrations, a preserved Ang I/Ang II ratio, and prevention of AT_1_ receptor downregulation in the left ventricle. Moreover, the reduction in catecholamine exposure, myocardial injury and local tissue inflammation with PCZ administration may improve gas exchange during septic shock.

### Supplementary Information

Supplementary Material 1.

## Data Availability

The datasets used during the current study are available from the corresponding author on reasonable request.
