# Supplementary material for: Inhibition of circulating dipeptidyl-peptidase 3 by procizumab in experimental septic shock reduces catecholamine exposure and myocardial injury
Source: Intensive Care Med Exp. 2024 Jun 7;12:53. doi: 10.1186/s40635-024-00638-3 (PMC11161450; doi:10.1186/s40635-024-00638-3)
Supplement: Supplementary file 1 — Supplementary Material 1. [file 40635_2024_638_MOESM1_ESM.docx]

**SUPPLEMENT**

**Inhibition of Circulating Dipeptidyl-Peptidase 3 by Procizumab in Experimental Septic Shock Reduces Catecholamine Exposure and Myocardial Injury**

Bruno GARCIA^1,2^; Benoit TER SCHIPHORST^1,2^; Karine SANTOS^3;^ Fuhong SU^1^; Laurence DEWACHTER^4^; Francisco VASQUES-NÓVOA^5^; Estela ROCHA-OLIVEIRA^5^; Roberto RONCON-ALBUQUERQUE Jr.^5^; Theo IKENNA UBA^3^, Oliver HARTMANN^3^; Adrien PICOD^6^; Feriel AZIBANI^6^; Jacques CALLEBERT^6,7^; Serge GOLDMAN^8^; Filippo ANNONI^1,9^; Raphaël FAVORY^2^; Jean-Louis VINCENT^1,9^; Jacques CRETEUR^1,9^; Fabio Silvio TACCONE^1,9^; Alexandre MEBAZAA^6,10^; Antoine HERPAIN^1,11^

^1^Experimental Laboratory of the Department of Intensive Care, Université Libre de Bruxelles (ULB), Brussels, Belgium

^2^Department of Intensive care, Centre Hospitalier Universitaire de Lille, Lille, France

^3^4TEEN4 Pharmaceuticals GmbH, Hennigsdorf, Germany

^4^Laboratory of Physiology and Pharmacology, Université Libre de Bruxelles (ULB), Brussels, Belgium

^5^Cardiovascular R&D Center, Faculty of Medicine, University of Porto, Porto, Portugal

^6^Université Paris Cité, UMR-S 942, INSERM, MASCOT, Paris, France

^7^Department of Biochemistry, Assistance Publique Hôpitaux de Paris, Hôpital Lariboisière, Paris, France

^8^Department of Nuclear Medicine, Hôpital Universitaire de Bruxelles (HUB), Université Libre de Bruxelles (ULB), Brussels, Belgium

^9^Department of Intensive Care, Hôpital Universitaire de Bruxelles (HUB), Université Libre de Bruxelles (ULB), Brussels, Belgium

^10^Department of Anesthesia, Burn and Critical Care, University Hospitals Saint-Louis- Lariboisière, AP-HP, Paris, France

^11^Department of Intensive Care, Saint-Pierre University Hospital, Université Libre de Bruxelles (ULB), Brussels, Belgium

**CORRESPONDING AUTHOR:** Bruno GARCIA, MD, MSc; Experimental Laboratory of the Department of Intensive Care, Université Libre de Bruxelles (ULB), Brussels, Belgium.

E-mail: [br.garcia@icloud.com](mailto:br.garcia@icloud.com)

**METHODS**

**Experimental protocol**

Animals were fasted for 18 hours prior to the start of the experiment with free access to water. Thereafter, they were sedated in their enclosure with an intramuscular injection of midazolam (1 mg/kg) and ketamine hydrochloride (20 mg/kg) in the neck. After transportation to the operating room, a peripheral line was placed in a vein of the ear and a 4.5 F arterial catheter (Terumo Medical Company, Belgium) was placed in the left common femoral artery for invasive monitoring of arterial pressure and blood sampling. Following anesthesia induction with an intravenous injection of 3 μg/kg of sufentanyl, 1 mg/kg of propofol and 0.5 mg/kg of rocuronium, endotracheal intubation was performed; general anesthesia was achieved with continuous inhalation of sevoflurane (at 1.8 to 2.5% alveolar concentration) and analgesia with a continuous infusion of morphine (0.2 to 0.5 mg/kg*h), in association with rocuronium (1.8 to 2.0 mg/kg/h); the optimal dose of analgo-sedation being determined through repeated pain tests, i.e., change in heart rate or blood pressure after nasal septum pinching. Volume-controlled mechanical ventilation (Primus, Draëger, Lübeck, Germany) was used with a fixed tidal volume of 8 mL/kg, a positive end-expiratory pressure of 5 cmH_2_O, a fraction of inspired oxygen (FiO_2_) adjusted to keep PaO_2_ >90 mmHg, and a respiratory rate adjusted to maintain an arterial pH between 7.35 and 7.45. For drug infusion, a three-lumen central venous catheter (Terumo Medical Company, Belgium) was inserted percutaneously into the right external jugular vein under ultrasound guidance (Vivid E90, GE Machines, USA).

A pulmonary artery catheter (CCO, Edwards LifeSciences, Irvine, California, USA) was advanced through the left external jugular vein into the pulmonary artery for measurement of right heart pressures and continuous monitoring of cardiac output (CO) and mixed venous oxygen saturation (Svo_2_). The electrocardiogram, intravascular pressures and CO were continuously displayed (SC9000, Siemens, Munich, Germany) and exported to an A/D recording station (Notocord-Hem 4.4, Notocord, France). A pressure sensor catheter (Millar® 5F Pressure Catheter, Texas, USA) was introduced in the right common femoral artery. Arterial pulse pressure variation (PPV) was automatically calculated from the arterial femoral signal using the formula “PPV = PP_max_ – PP_min_ / (PP_max_ + PP_min_) / 2”, with PP being the pulse pressure (i.e., the difference between systolic and diastolic arterial pressures). A left ventricular (LV) pressure catheter (5 Fr, Transonic® Europe BV, Elsloo, The Netherlands) was inserted into the left ventricle through the internal carotid artery and was connected to an ADV500 system (Transonic® Europe BV).

Fluid maintenance was achieved using a balanced crystalloid solution (Plasmalyte, Baxter, USA) at a perfusion rate of 5 to 10 mL/kg.h, aiming to maintain the PPV ≤ 13% when mean arterial pressure decreased. Hypoglycemia was avoided by continuous infusion of a 20% glucose solution (1 to 2 mL/kg.h). A 14Fr Foley catheter was surgically introduced into the bladder via a supra-pubic mini-laparotomy to monitor urine output. Finally, two abdominal drains were placed on each side of the abdominal cavity for the later introduction of autologous feces and peritoneal lavage.

Four animals (sham operated group), which underwent only anesthesia and surgical preparation, were observed for 4 hours after baseline until euthanasia and were used as a reference for tissue analysis.

**Procizumab (PCZ) infusion**

In the interventional group, once the shock time point was reached, a continuous infusion of PCZ was started at 9.3 mg/kg. PCZ, an G1 immunoglobulin inhibiting cDPP3 activity, was generated as previously described [1] in Chinese hamster ovary cells and purified via Protein A chromatography followed by a polishing step consisting of an anion-exchange membrane with a consecutive multi-modal chromatography step. The antibody underwent ultra- and diafiltration during which it was concentrated to 20 mg/mL and formulated into its final formulation buffer. The antibody solution was placed under aseptic conditions into 20R DIN glass vials, labelled, packed, and provided by 4TEEN4 Pharmaceuticals GmbH (Hennigsdorf, Germany). PCZ was stored at 2-8 °C under temperature control.

**Procizumab concentration measurement**

The PCZ concentration was measured at Eurofins laboratories using an assay developed by 4TEEN4 Pharmaceuticals GmbH. Briefly, the method consists of a sandwich ELISA. First, each well was coated with 100 µL of a solution of anti-PCZ antibody at a concentration of 0.5 µg/mL. After overnight incubation, the wells were blocked with 300 µL of superblock blocking buffer in PBS. Calibrators and quality control samples were prepared in neat serum and applied with minimum required dilution (MRD) of 1/20 in buffer. Following sample addition, PCZ was captured and detected adding 100 µL per well of a solution of anti-human IgG (Fc specific)-peroxidase-linked antibody at a concentration of 0.1 µg/mL. Finally, the 3,3′,5,5′-Tetramethylbenzidine (TMB) substrate was added and converted by the peroxidase linked to the detector antibody. This enabled measurement of a colorimetric signal that is directly proportional to the concentration of PCZ. The specificity of the method was tested to attest its ability to quantify solely and specifically PCZ in the presence or absence of DPP3 (drug target). The analysis was performed using a 4-parameter-logistic regression model.

**Biological measurements**

Arterial and venous blood gas measurements were taken respectively from femoral and pulmonary artery blood samples, at each time point. These measurements were performed directly in the animal laboratory using a blood gas and electrolyte analyzer (Cobas b-123, Roche, Switzerland).

At each time point, the plasma samples were collected from the femoral artery and sent to a dedicated laboratory (Synlab Veterinary, Heppignies, Belgium) for analysis: blood count, platelets, urea, creatinine, aspartate aminotransferases, alanine transaminase, protein, albumin, and high-sensitivity cardiac troponin-I levels. Urine samples were analyzed by the same laboratory for urinary urea, creatinine, sodium and potassium concentrations.

For analysis of cytokine, catecholamine and cDPP3 concentrations, centrifugation was performed directly in the animal laboratory at a fixed temperature of 4°C and 2500 rotations per minute for a duration of 15 minutes (SL 8R, ThermoFisher Scientific, Germany).

EDTA tubes for DPP3 activity analyses were centrifuged and then immediately frozen at -80 °C. Heparin tubes for equilibrium angiotensin system analysis were centrifuged immediately, kept at room temperature for 30 minutes, and then frozen.

**Enzyme-linked immunosorbent assay (ELISA)**

Plasma concentrations of interleukin (IL)-6, IL-10, and tumor necrosis factor (TNF)-α were determined using Quantikine Porcine IL-6, IL-10 and TNF-α ELISA kits (R&D system, Minneapolis, USA), respectively, according to the manufacturer’s protocols.

**DPP3 activity measurements**

The DPP3 activity was measured at 4TEEN4 Pharmaceuticals GmbH, Germany as previously described [1]. Briefly, the inhibitory potential of PCZ was measured in a standard soluble activity assay (SAA) with swine samples and a fluorogenic substrate. A 10 µL of sample was incubated with 90 µL of a substrate reagent solution (50 mM Tris/HCl, pH 7.8 (25°C), 0.125% Triton X-100, 0.125 mM Arg-Arg-β-napthylamide (Arg2-βNA), 1 mM CoCl_2_ and 0.25 mM amastatin) for 1 h at 37°C in black 96-well microtiter plates. Fluorescence of the cleavage product βNA was detected using the Twinkle LB 970 fluorometer (Berthold Technologies GmbH) with an excitation wavelength of 340/10 nm and emission wavelength of 420/10 nm. Immediately after sample pipetting, baseline fluorescence (t=0 min) was measured. The plate was covered and incubated for an hour at 37 °C in the dark without agitation. Finally, the fluorescence was measured at t=60 min and corrected for baseline signal. The fluorescence values of the calibrator were plotted against their respective known activities. Based on this linear standard curve, the sample’s fluorescent signals were converted into measures of enzymatic activity.

**Catecholamine measurements**

Circulating epinephrine, norepinephrine and dopamine were measured after EDTA plasma sample preparation through solid phase extraction (Chromsystems Instruments, Gräfelfing, Germany), using an isocratic high performance liquid chromatography system with electrochemical detection (Coularray®, ESA, Chelmsford, United States).

**Renin angiotensin system equilibrium analysis by liquid chromatography–tandem mass spectrometry**

Equilibrium angiotensins concentrations were determined by mass spectrometry via LC-MS/MS using heparin plasma samples as previously described [2] (Attoquant Diagnostics, Vienna, Austria).

**Nuclear medicine assessment**

The injectable radiopharmaceutical formulation used consisted of human serum albumin microaggregates (Pulmocis, Curium Pharma, Paris, France), with a median size of 30 µm (90% confidence interval 15-60µm) and labeled with technetium-99m (99mTc). Just after the H12 time-point, albumin microaggregates labelled with a load of 200 MBq of ^99m^TC were injected into the left ventricle cavity, over 30 seconds, using a 5F catheter (Cordis, Miami Lakes, USA) with multiple holes. Arterial blood was simultaneously withdrawn from the left femoral artery over 90 seconds, at a fixed rate of 15 mL/min, using an electrical pump (Pump 11 Elite, Harvard Apparatus, USA). The collected blood volume in the syringe and the different tissue biopsies (five tissue samples from the right kidney medulla and five from the cortex, along with five samples from the ileum) were then weighed. The respective ^99m^Tc activity of the blood in all these samples was measured by the laboratory of nuclear medicine of Erasme hospital. Thereafter, the respective organ perfusions of the renal cortex, the renal medulla and the ileum were calculated, as previously described with radioactive microspheres [3].

**Autopsy**

Upon completion of the experiment, the animals were euthanized by a 40 mL injection of 7.5% potassium chloride while under deep anesthesia. Autopsies were promptly carried out. Vascular samples from the aorta, right femoral and radial arteries were stored in RNA later solution (Invitrogen™, RNAlater™ Stabilization Solution, ThermoFisher Scientific, MA, USA) for mRNA expression analysis, and were flash frozen in liquid nitrogen for protein analysis.

**Real-time quantitative polymerase chain reaction (RTq-PCR)**

Total RNA was extracted from snap-frozen vascular tissue stored at -80°C in RNA later solution (Invitrogen™, *RNA later*™ Stabilization Solution, ThermoFisher Scientific, MA, USA), using a RNeasy Mini kit (QIAGEN, Germany). RNA concentration was determined by a standard spectrophotometric technique, using a Nanodrop® ND-1000 (Isogen Life Science, Netherlands). RNA integrity was assessed by visual inspection of GelRed (Biotium, California)-stained agarose gels. Reverse transcription was performed using random hexamer primers and Superscript II Reverse Transcriptase (Invitrogen, Carlsbad, USA), according to the manufacturer’s instructions. For RTq-PCR, sense and anti-sense primers were designed using the Primer3 program for *sus scrofa*  IL-6 RNA sequences (Table S1). To avoid inappropriate amplification of residual genomic DNA, intron-spanning primers were selected when exon sequences were known. For each sample, the ampliﬁcation reaction was performed in triplicate using SYBRGreen PCR Master Mix (Quanta Biosciences, Gaithersburg, MD), speciﬁc primers, and diluted template complementary DNA using an iCycler system (BioRad Laboratories). Relative quantification was achieved using the comparative 2^-ΔΔCt^ method by normalization with the housekeeping gene (beta-actin). Results are expressed as relative fold increase above the mean value of vascular relative mRNA expression of the sham operated group arbitrarily ﬁxed at 1.

**Table S1 Primers used for real-time quantitative polymerase chain reaction (RTQ-PCR) in porcine myocardial and vascular tissue.**

**Alpha1 – adrenergic receptor**

Sense 5'- AGTGATGCCCATTGGGTCTTT-3'

Antisense 5'- ATGGGGTTGATGCAGCTGTT-3'

**Beta 1 - adrenergic receptor**

Sense 5'- ACCCCAAGTGCTGCGATTT-3'

Antisense 5'- ATGCACAAGGGCACGTAGAA-3'

**Beta 2 – adrenergic receptor**

Sense 5'- GATTCACAGGGGAGGAACTGTAG-3'

Antisense 5'- TTGTTTAGTGTTTGGCTGGGAG-3'

**Angiotensin II receptor type 1 (AT1)**

Sense 5'- ACCCAAACCCCATACCAGAG-3'

Antisense 5'- AAAACCCGGCCATTTTAGAC-3'

**Angiotensin II receptor type 2 (AT2)**

Sense 5'- CCTGACCCTGAACATGTTTGC-3'

Antisense 5'- GAGGCTTGCCAGGGATTTCT-3'

**Interleukin-6 (IL-6)**

Sense 5'- CCACCAGGAACGAAAGAGAG -3'

Antisense 5'- AGTAGCCATCACCAGAAGCAG -3'

**Immunoblotting**

Immunoblotting was performed as previously described [4,5]. Briefly, 40μg of protein were separated by SDS-PAGE using 4-20% gradient polyacrylamide gels (Criterion™ TGX™ Precast Gels, #5671095, Bio-Rad) and then electroblotted into nitrocellulose membranes (Trans-Blot® Turbo, Bio-Rad). Blots were blocked and incubated with primary antibodies (Table S2) overnight at 4ºC. The immunoblots were subsequently washed and incubated with infra-red dye-conjugated antibodies (LI-COR Biosciences; Table E2). Protein phosphorylation status was evaluated incubating the membrane simultaneously with host mismatched primary antibodies targeting total and phosphorylated forms, which were identified with different fluorochrome-coupled secondary antibodies. The membrane was imaged by scanning at both 800 and 700 nm with Odyssey Infrared Imaging System (LICOR Biosciences). GAPDH was used as internal control and the control group was set as reference.

**Table S2 Primary antibodies used for immunoblot.**

| PRIMARY ANTIBODIES | | | |
| --- | --- | --- | --- |
| Protein | **Isotype** | **Source** | **Reference** |
| AT_1_ | Rabbit IgG | Sigma-Aldrich | AB15552 |
| AT_2_ | Rabbit IgG | Abcam | ab92445 |
| $\boldsymbol{\alpha}$_1_AR | Rabbit IgG | Abcam | ab137123 |
| $\boldsymbol{\beta}$_1_AR | Rabbit IgG | Abcam | ab3442 |
| $\boldsymbol{\beta}$_2_AR | Rabbit IgG | Abcam | ab182136 |
| GAPDH | Mouse IgG1 | Abcam | ab8245 |
| SECONDARY ANTIBODIES | | | |
| Antibody | **Isotype** | **Source** | **Reference** |
|  | Goat | LI-COR |  |
| IRDye® 680LT anti-Mouse | Goat | LI-COR |  |

**AT_1_** - Angiotensin II type 1 receptor; **AT_2_** - Angiotensin II type 2 receptor; $\alpha$**_1_AR** - Alpha 1 adrenergic receptor; $\beta$**_1_AR** - Beta 1 adrenergic receptor;; $\beta$**_2_AR** - Beta 2 adrenergic receptor; ; **GAPDH** - Glyceraldehyde-3-Phosphate Dehydrogenase

**RESULTS**

**Figure S1 Relative changes in creatinine levels from baseline (%)**

Values are shown as median and interquartile range. P-values for interaction were analyzed using a generalized linear model, n=8 for sham operated group, n=8 for PCZ.

**
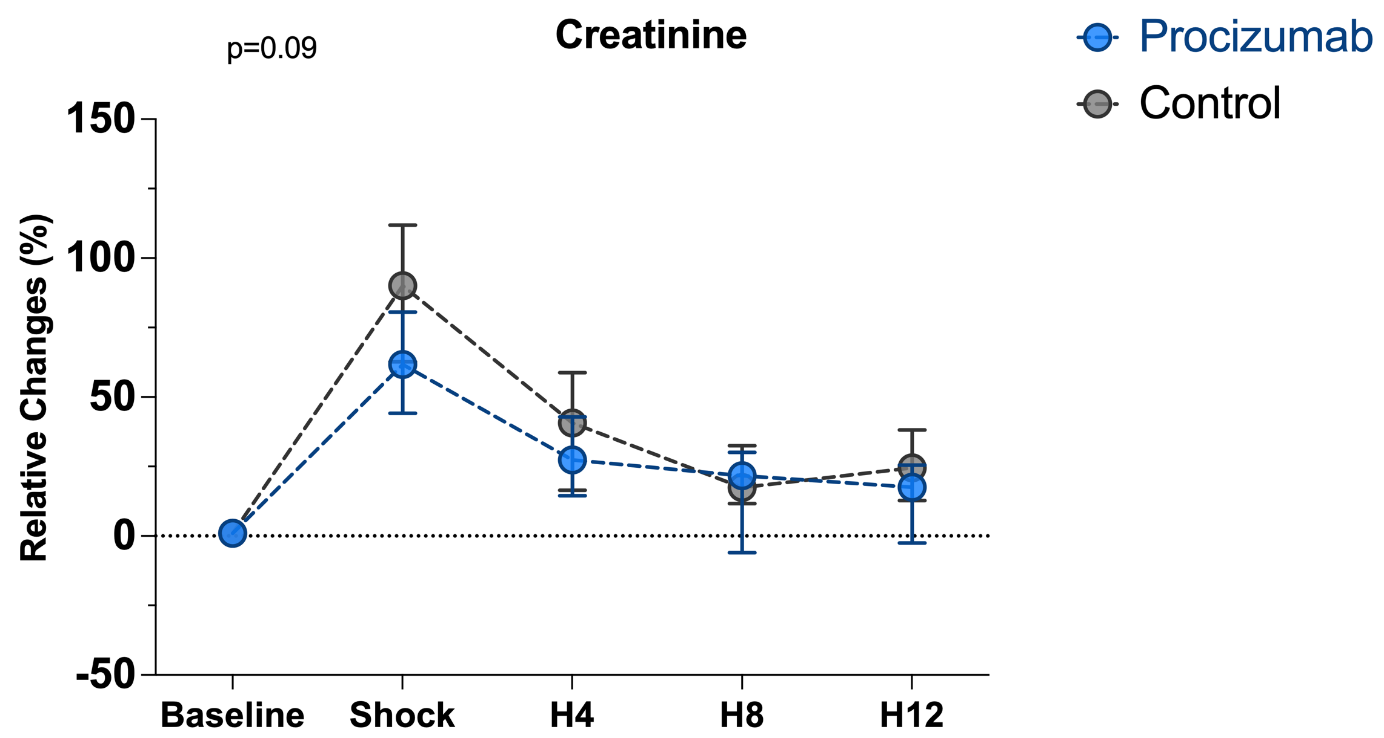
**

**Figure S2: Pulse pressure variation and left ventricle end diastolic pressure**

Indexes of cardiac preload (pulse pressure variation and left ventricle end diastolic pressure).

Values are expressed as median – interquartile range. BL: baseline; S: shock time-point; R: Resuscitation time-point.

P-values for interaction were analyzed using a generalized linear model, n=8 for sham operated group, n=8 for PCZ.

**
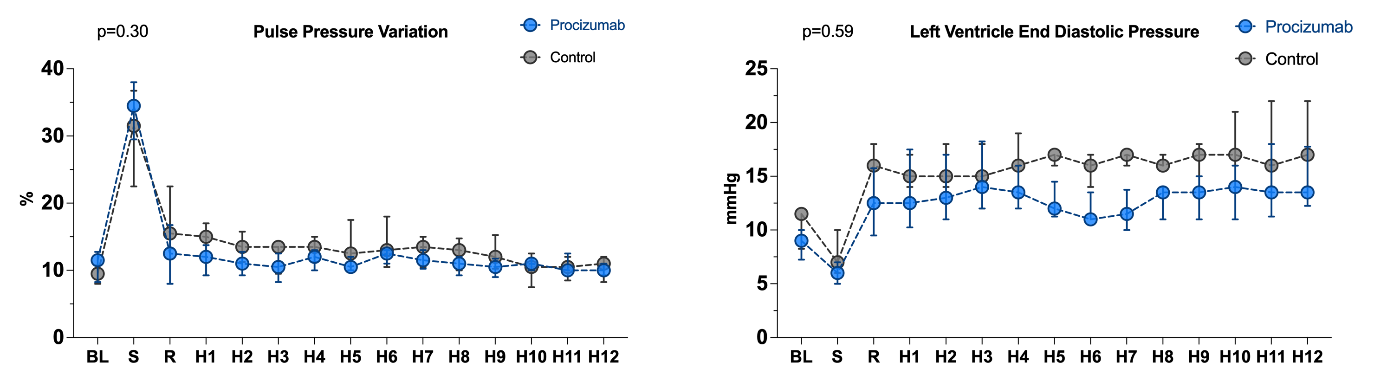
**

**Figure S3: Evolution of dipeptidyl peptidase 3 and procizumab concentrations over time in the two groups.**

Values are expressed as mean +/- standard deviation**.**

P-value for interaction between groups for cDPP3 measurements. P-values for interaction were analyzed using a generalized linear model. *p-value<0.05 for post hoc analysis, n=8 for sham-operated group, n=8 for PCZ.

R: Resuscitation time point, after one hour of hypoperfusion


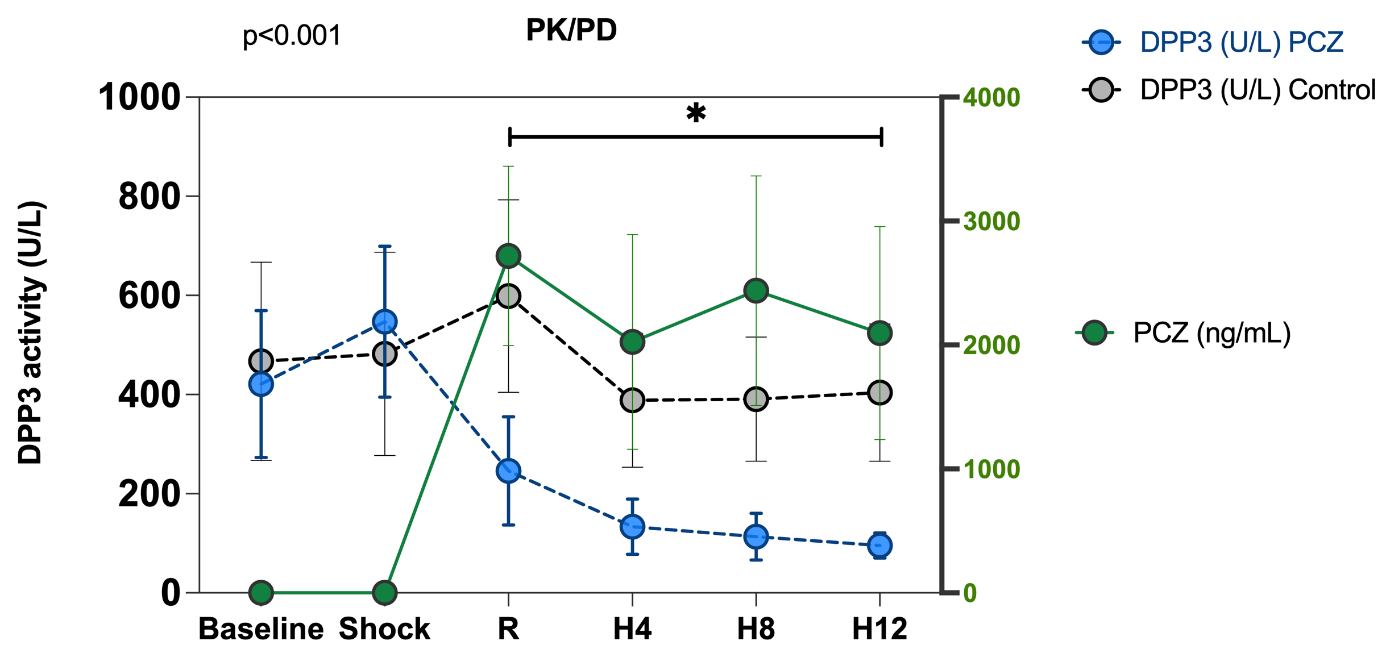


**FIGURE S4: IL-6 expression, myocardial and vascular angiotensin receptors**

1. Relative mRNA expression of IL-6 in the left ventricle (n=3 for sham operated group, n=6 for control septic, n=7 for PCZ), aorta (n=4 for sham operated group, n=8 for control, n=8 for PCZ), femoral (n=4 for sham operated group, n=7 for control septic group, n=8 for PCZ) and radial (n=4 for sham operated group, n=5 for control septic, n= 6 for PCZ) arteries.

Relative quantification was achieved using the comparative 2^−ΔΔCt^ method by normalization with the housekeeping gene (ActB‑actin).

Results are expressed as relative fold increase above the mean value of relative mRNA expression of the sham group arbitrarily fixed at 1.

1. Relative mRNA expression of AT_1_ and AT_2_ mRNA (n=4 for sham operated group, n=8 for control septic group, n=8 for PCZ) and relative protein expression (n=4 for sham operated group, n=7 for control, n=8 for PCZ) in the left ventricle. Results are expressed as relative fold increase above the mean value of the sham group arbitrarily fixed at 1.
2. AT_1_ and AT_2_ mRNA and protein expression in the aorta (n=4 for sham operated group, n=7 for control septic group, n=8 for PCZ), femoral (n=4 for sham operated group, n=6 for control septic group, n=8 for PCZ) and radial (n=4 for sham operated group, n=7 for control septic group, n=8 for PCZ) arteries. Results are expressed as relative fold increase above the mean value of the sham group arbitrarily fixed at 1.

Values are expressed as median & interquartile range. *****P-value <0.05 for the Kruskal-Wallis test between PCZ and control septic group.

*****P-value<0.05 ; ** P-value<0.01 between PCZ and control septic group.

**
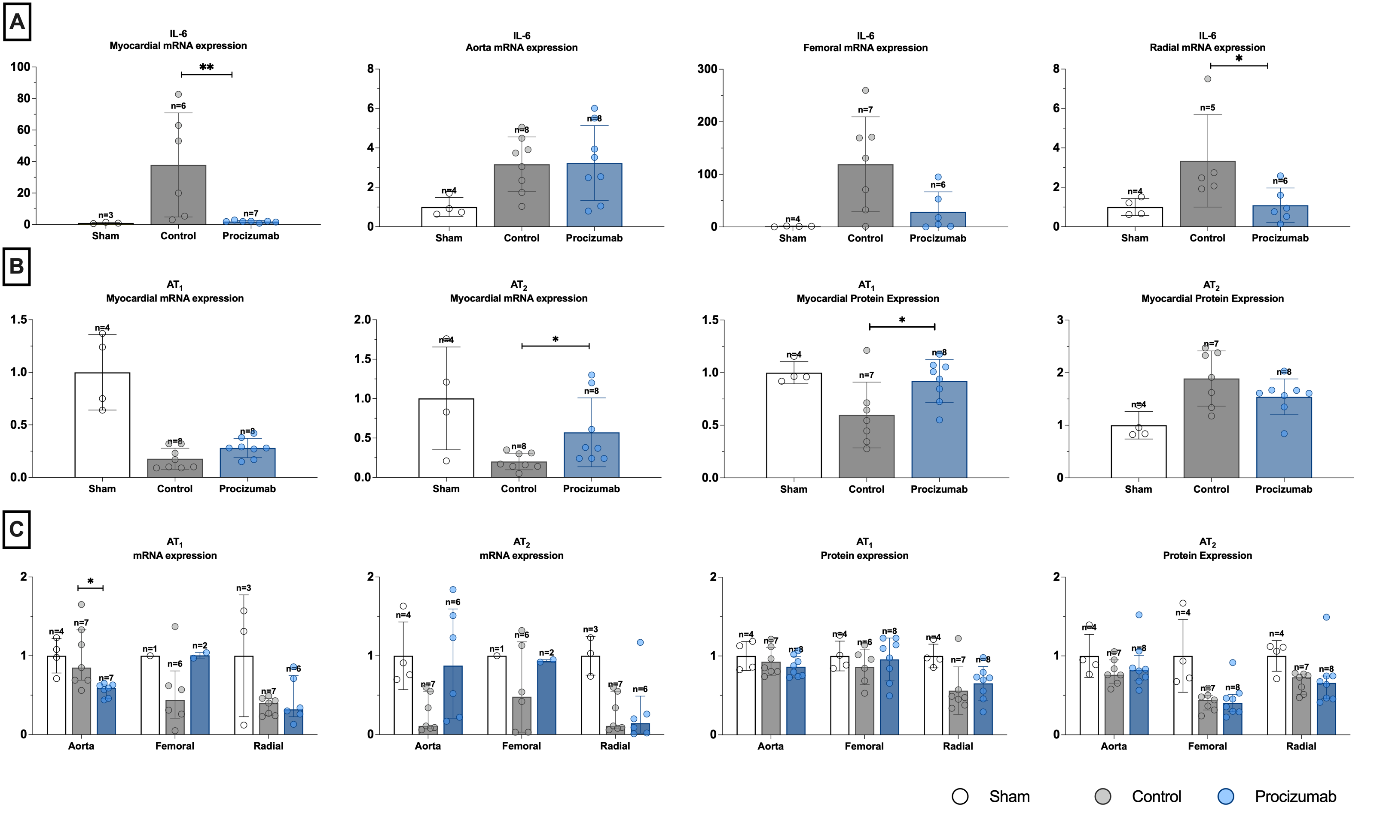
**

**FIGURE S5: Myocardial and vascular mRNA and protein expression of adrenergic receptors**

1. Relative mRNA expression of alpha-1, beta 1 and beta-2 adrenergic receptors in the left ventricle (n=4 for sham operated group, n=8 for control septic group, n=8 for PCZ), aorta (n=3 for sham operated group, n=7 for control septic group, n=8 for PCZ), femoral (n=1 for sham operated group, n=5 for control septic group, n=2 for PCZ), and radial (n=3 for sham operated group, n=7 for control septic group, n=6 for PCZ) arteries.

Relative quantification was achieved using the comparative 2^−ΔΔCt^ method by normalization with the housekeeping gene (ActB‑actin).

Results are expressed as relative fold increase above the mean value of relative mRNA expression of the sham operated group arbitrarily fixed at 1.

1. Alpha-1, beta-1 and beta-2 adrenergic receptor mRNA and protein expression in the left ventricle, aorta, femoral and radial arteries (n=4 for sham operated group, n=7 for control septic group, n=8 for PCZ). Results are expressed as relative fold increase above the mean value

Values are expressed as median & interquartile range

*****P-value <0.05 for the Kruskal-Wallis test between PCZ and control septic group.

* P-value<0.05


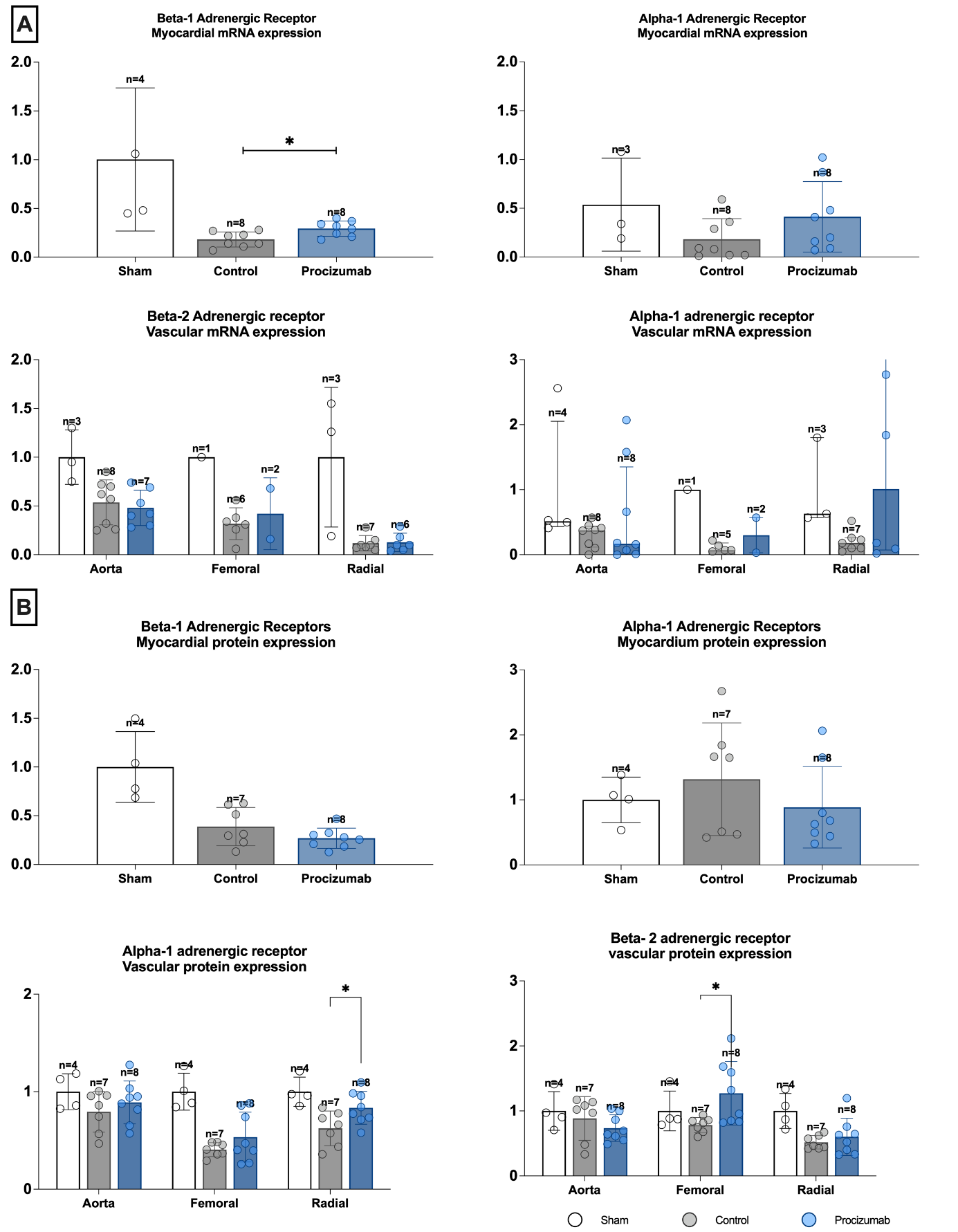


**Figure S6: Myocardial and vascular representative blots of angiotensin II receptor 1 (AT_1_)**


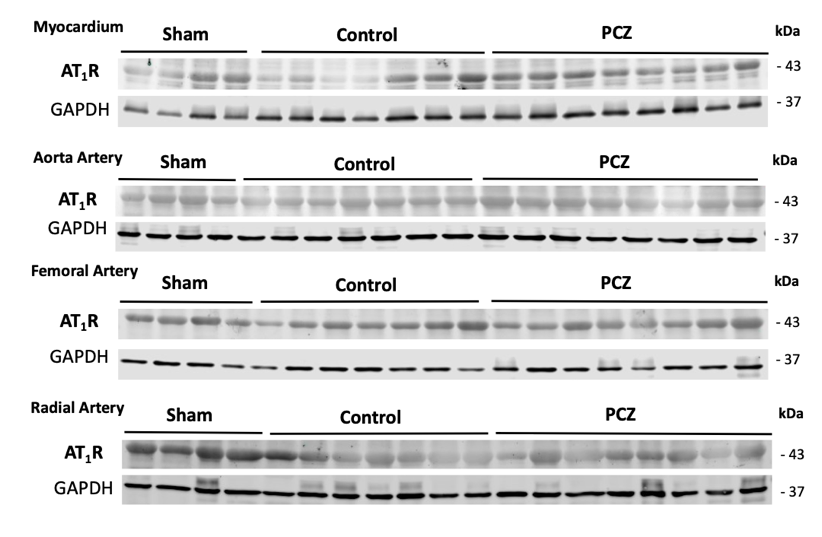


**Figure S7: Myocardial and vascular representative blots of angiotensin II receptor 2 (AT_2_)**


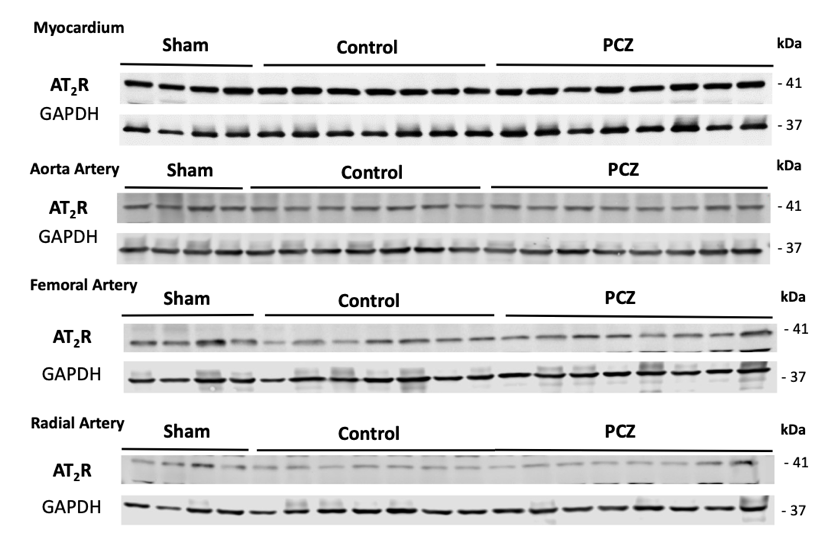


**Figure S8: Myocardial and vascular representative blots of adrenergic receptors**

**Figure S9: Vascular representative blots of Beta-2 adrenergic receptors**

**Table S3: Hemodynamic variables in the two groups at the different study time-points**

Values are expressed as median ± interquartile range

P-values for interaction were analyzed using a generalized linear model. *P-value for time point differences

SV: stroke volume; RAP: right atrial pressure: PAWP: Pulmonary artery wedge pressure

| VARIABLES  Mean +/- SD |  | Baseline | Shock | H4 | H8 | H12 | P-value |
| --- | --- | --- | --- | --- | --- | --- | --- |
| SV  (ml/kg) | PCZ Control | 1.2 [1-1.3]  1.1 [1-1.3] | 0.4 [0.3-0.4]  0.4 [0.3-0.4] | 1.1 [1.1-1.2]  1.4 [1.1-1.7] | 1.1 [0.9-1.2] *****  1.5 [1.2-1.7] | 1.2 [1-1.3] *****  1.4 [1.3-1.6] | 0.02 |
| RAP  (mmHg) | PCZ Control | 8 [7-9]  9 [6-11] | 4 [1-7]  4 [3-5] | 6 [5-10]  8 [7-12] | 7 [5-10]  10 [7-11] | 9 [6-10]  9 [6-12] | 0.41 |
| PAWP  (mmHg) | PCZ Control | 8 [4-12]  7 [6-10] | 6 [5-10]  3 [2-6] | 10 [6-13]  10 [7-13] | 12 [8-13]  9 [8-13] | 14 [11-14]  10 [9-15] | 0.09 |

**Table S4: Transaminase evolution in the two groups at the different study time points**

Values are expressed as median & interquartile range

P-values for interaction were analyzed using a generalized linear model. * P-value<0.05 for time-point differences

|  |  | Baseline | Shock | H4 | H8 | H12 | p-value |
| --- | --- | --- | --- | --- | --- | --- | --- |
| Aspartate transaminase  (UI/L) | PCZ  Control | 33 [26-36]*  57 [50-61] | 48 [39-83]  74 [61-78] | 83 [57-108]  78 [60-171] | 114 [87-225]  90 [75-163] | 121[113-282]  102 [76-163] | 0.0183 |
| Alkaline phosphatase  (UI/L) | PCZ  Control | 45 [39-48]  48 [42-55] | 46 [33-48]  47 [41-53] | 24 [19-32]  21 [18-39] | 28 [19-41]  28 [19-40] | 31 [22-48]  29 [20-51] | 0.93 |

**REFERENCES**

1. Deniau B, Rehfeld L, Santos K, Dienelt A, Azibani F, Sadoune M, et al. Circulating dipeptidyl peptidase 3 is a myocardial depressant factor: dipeptidyl peptidase 3 inhibition rapidly and sustainably improves haemodynamics. Eur J Heart Fail. 2020;22:290–9.

2. Reindl-Schwaighofer R, Hödlmoser S, Eskandary F, Poglitsch M, Bonderman D, Strassl R, et al. Angiotensin-Converting Enzyme 2 (ACE2) Elevation in Severe COVID-19. Am J Resp Crit Care. 2021;

3. Buckberg GD, Luck JC, Payne DB, Hoffman JI, Archie JP, Fixler DE. Some sources of error in measuring regional blood flow with radioactive microspheres. J Appl Physiol. 1971;31:598–604.

4. Vasques-Nóvoa F, Laundos TL, Cerqueira RJ, Quina-Rodrigues C, Soares-dos-Reis R, Baganha F, et al. MicroRNA-155 Amplifies Nitric Oxide&sol;cGMP Signaling and Impairs Vascular Angiotensin II Reactivity in Septic Shock. Crit Care Med. 2018;46:e945–54.

5. Garcia B, Su F, Dewachter L, Favory R, Khaldi A, Moiroux-Sahraoui A, et al. Myocardial effects of angiotensin II compared to norepinephrine in an animal model of septic shock. Crit Care. 2022;26.
